# A great way to bring up health behaviour topics at playgroup: a qualitative evaluation of the Healthy Conversations @ Playgroup program

**DOI:** 10.1186/s12889-024-17703-x

**Published:** 2024-03-25

**Authors:** Georgia Middleton, Brittany J. Johnson, Dimity Dutch, Stewart G. Trost, Rebecca Byrne, Hayley E. Christian, Anna Henry, Caroline O. Terranova, Kate E. Williams, Li Kheng Chai, Denise S. K. Brookes, Kate Simon, Rebecca K. Golley

**Affiliations:** 1 Flinders University, College of Nursing and Health Sciences, Caring Futures Institute, Adelaide, SA Australia; 2 School of Human Movement and Nutrition Sciences, The University of Queensland Australia, Brisbane, QLD Australia; 3 Faculty of Health, School of Exercise and Nutrition Science, Queensland University of Technology, Brisbane, QLD Australia; 4 Telethon Kids Institute, The University of Western Australia, Perth, WA Australia; 5 School of Population and Global Health, The University of Western Australia, Perth, WA Australia; 6 Centre for Child and Family Studies, School of Early Childhood and Inclusive Education, Queensland University of Technology, Brisbane, QLD Australia; 7 Health and Wellbeing Queensland, Queensland Government, Brisbane, QLD Australia

**Keywords:** Early childhood, Community, Parenting, Peer-led program, Healthy eating, Movement, Sleep, Screentime, Playgroup

## Abstract

**Background:**

The early years is a critical stage to establish optimal nutrition and movement behaviours. Community playgroups are a relaxed environment for parents with a focus on social connection and supporting parents in their role as ‘First Teachers’. Playgroups are therefore an opportunistic setting to promote health behaviours in the early years. To support parents with young children around healthy lifestyle behaviours, the *Healthy Conversations @ Playgroup* program was delivered in urban and regional areas, across three Australian jurisdictions between 2021–2023.

**Objective:**

This qualitative evaluation aimed to understand how the *Healthy Conversations @ Playgroup* program was experienced by parents, playgroup coordinators and peer facilitators.

**Design:**

Semi-structured virtual interviews and focus groups were conducted with parents, playgroup coordinators (i.e., person responsible for coordinating the playgroup) and peer facilitators (i.e., trained facilitator for the program) that participated in the *Healthy Conversations @ Playgroup* study. Transcripts were analysed following a thematic analysis approach.

**Results:**

Twenty-eight playgroup parents, coordinators or peer facilitators participated in one of 8 focus groups or 5 interviews. Four themes were developed: Program strengths and challenges; Setting strengths and challenges; Factors that impact program delivery; Participant’s suggestions for future program delivery.

**Conclusions:**

The *Healthy Conversations @ Playgroup* program was valued by parents, providing validation and normalisation of parenting practices, and fostering a shared experience of parenting. Playgroups are a convenient setting for families to attend. The dynamic and distracting nature of the playgroup setting were carefully considered when designing the program. Strategies to further enhance program engagement could include use of coordinator or parent champions, tailored delivery, and extending the reach to other family members.

**Trial registration:**

Australian New Zealand Clinical Trials Registry ACTRN12621000055808, registered 22 January 2021, https://www.anzctr.org.au/Trial/Registration/TrialReview.aspx?id=380890

**Supplementary Information:**

The online version contains supplementary material available at 10.1186/s12889-024-17703-x.

## Background

The early years of life are critical for establishing health-promoting behaviours to support optimal health, growth, and development [1, 2]. Health-promoting behaviours include regular physical activity, limited screen time, healthy eating, and adequate sleep [1]. However, recent population-level surveys indicate that only 28% of Australian children aged 2–3 years are meeting both fruit and vegetable recommendations [3], and only 17% of Australian children aged 2–5 years are meeting both physical activity and sedentary behaviour recommendations [4]. Health behaviours established in the early years can track into adolescence and adulthood, influencing health across the life course [2, 5, 6]. Therefore, it is important to intervene early and establish healthy behaviours in childhood [7–11].

Parents are children’s first teachers, and their parenting practices are instrumental in shaping children’s eating, movement, and sleep behaviours [12–14]. Parenting practices are specific, observable parenting actions such as creating a safe, interesting environment, setting limits and rules, having realistic expectations, and using appropriate feedback and consequences [15, 16]. Supportive parenting practices and the family environment are integral for developing child autonomy [17]. Autonomy supporting parenting practices, where parents encourage thoughtful child decision-making, have been shown to support the development of healthy behaviours in children [18]. However, previous research has indicated that parents require knowledge, skills, and confidence to effectively use autonomy supportive practices to promote child health behaviours [14, 19].

Parent involvement has been recognised as integral for improving child health behaviour outcomes that support healthy growth [20–22]. However, programs delivered through Early Childhood Education and Care settings, while suitable for reaching a large proportion of preschool-aged children, are not necessarily conducive to parental participation and engagement as parents time at the setting is limited [23, 24]. Programs delivered in community settings where parents already attend with their child may have a higher likelihood of success, particularly where there are existing mechanisms for parent support [25]. Community playgroups are one such setting, offering a unique model of informal family support by bringing together groups of families with young children in local settings for shared play and socialising. Community playgroups offer a low- or no-cost, safe, and relaxed environment where existing social networks exist among attending parents, they enable shared learning and support, and are facilitated by a playgroup coordinator who is often a parent volunteer [26]. Despite playgroups existing internationally, including in the United Kingdom and United States, few child health promotion programs have been delivered and evaluated in community settings such as playgroups [27].

In 2018, Fuller and colleagues conducted focus groups with parents attending community playgroups in Brisbane, Australia, to determine what parents would find acceptable in a program delivered in playgroups [28]. The findings indicated that parents did not want to be ‘educated’ but desired strategies and support for dealing with parenting challenges. This aligns with previous reports that programs supporting parents commonly provide education, advice, and strategies [16, 29, 30] but parents also require support for increased capability and confidence [7, 28, 31, 32]. Additionally, parents did not want to lose their valuable playgroup time to an external program and felt the support and guidance received from other parents at playgroup facilitated autonomy supporting parenting practices [28].

The *Healthy Conversations @ Playgroup* program was designed to support parents to use autonomy promoting parenting practices to improve children’s eating, movement, screen time and sleep behaviours [26]. The program was designed to be suitable to embed in the universal care system. The program was evaluated in community playgroups, hereafter referred to as playgroups, in three Australian jurisdictions (South Australia, Western Australia, Queensland; urban and regional areas) as a multi-site randomised controlled trial (ACTRN12621000055808) [26]. Recruitment, program delivery and evaluation occurred between 2021–2023, over three waves (due to COVID-19). Playgroup associations in each state promoted the program to all registered playgroups, who self-selected to participate (*n* = 51 total playgroups participated in the evaluation). In brief, the H*ealthy Conversations @ Playgroup* program comprises 10 conversations delivered by a peer facilitator (a parent external to the playgroup, employed and trained to deliver the program) over five fortnightly sessions within the usual playgroup schedule [26]. The conversations were designed to increase parents’ capability and self-efficacy to implement autonomy-supportive parenting practices*.* Conversation topics included: reducing stress at mealtimes, limiting screens without tantrums, supporting movement skills in children, bedtime activities and routines to support sleep, and celebrating achievements. Further details of the program design and quantitative evaluation are reported in Trost et al. [26]. This qualitative study aimed to understand how the *Healthy Conversations @ Playgroup* program was experienced by parents, playgroup coordinators, and peer facilitators.

## Methods

### Study design

This study aligns with a critical qualitative approach, informed by critical realism ontology and an epistemological orientation of contextualism [33]. Through this position, we acknowledge that human practices shape the way we experience and know about reality and the world, and that human experiences cannot be studied in isolation from the contexts in which they exist [33]. This is well suited to understanding the shared experiences of participating in or delivering the *Healthy Conversations @ Playgroup* program. A thematic analysis approach guided the collection and analysis of data for this study [33, 34].

### Recruitment

Parents who participated in the *Healthy Conversations @ Playgroup* trial were eligible to participate in this qualitative study. As contact information of participating parents was collected for the broader program, this information was available to recruit parents into this qualitative study. Parents were invited via phone by a member of the research team to participate in a virtual focus group, between November 2022 to February 2023. Each playgroup in the program had a playgroup coordinator, a contact person who was typically a parent or community volunteer. Their contact information was also collected for the broader program and thus available to the research team to recruit into the qualitative study. Playgroup coordinators were invited via email or phone by a member of the research team to participate in a virtual focus group. Contact details of the peer facilitators who were responsible for delivering the *Healthy Conversations @ Playgroup* program were also available to the research team for this qualitative study. Peer facilitators were invited via email by a member of the research team to participate in a virtual one-on-one interview. Potential participants were provided with an information sheet, allocated to a suitable focus group or interview time, and asked to provide verbal (parents) or written consent (peer facilitators and playgroup coordinators) to participate.

### Data collection

Two semi-structured focus group/interview guides were developed, one for parents, and one for playgroup coordinators and peer facilitators (Additional file 1). Both guides were pilot tested with participants, and as they required no major changes their data were used in analysis. The guides were designed based on those used in Fuller et al.’s focus groups [28], and other qualitative explorations of parenting practices [35, 36]. The questions aimed to explore participants’ experiences of the program, what they perceived as program strengths and weaknesses, and what they would recommend for future iterations. Focus groups were chosen because they encourage group reflection and exploration of potentially sensitive issues by creating a safe space where similar experiences or views can be shared, and a shared experience can be created [37]. One-on-one interviews were chosen for the peer facilitators to encourage depth of responses, and to maintain their confidentiality as they were known to one another [37]. Peer facilitators were offered copies of their transcripts for review; none took up the offer. Due to the nature of focus groups, this was not possible for other participants.

All focus groups and interviews were conducted via video call using Microsoft Teams Version 1.6.00.11166, and were audio recorded and transcribed verbatim by professional transcription agency OutScribe Transcription, a human transcription service. GM (PhD), an experienced qualitative researcher, conducted all focus groups and interviews, and another member of the research team acted as notetaker (DD or research assistant). GM conducted the qualitative exploration as an independent party to the *Healthy Conversations @ Playgroup* program. They were not involved in the design, delivery, or evaluation of the program, and had no prior relationship to participants. This potentially helped reduce social desirability bias and protected participants from feeling pressured to provide a socially acceptable response to the designers or deliverers of the program.

The data collection and analysis team, comprising of GM, DD, BJJ, and a research assistant are white females with no children and approached this research from a background in public health and dietetics. All work in the space of child and family health and nutrition and have varying degrees of experience and knowledge working with this population group and researching childhood health behaviours and related parenting practices. GM had no prior experience with playgroups; however, BJJ, DD and the research assistant were involved in other aspects of the broader program, excluding program delivery. DD and the research assistant had minimal qualitative research experience prior to this study, but were supported and guided by GM.

### Data analysis

**Fig. 1:**
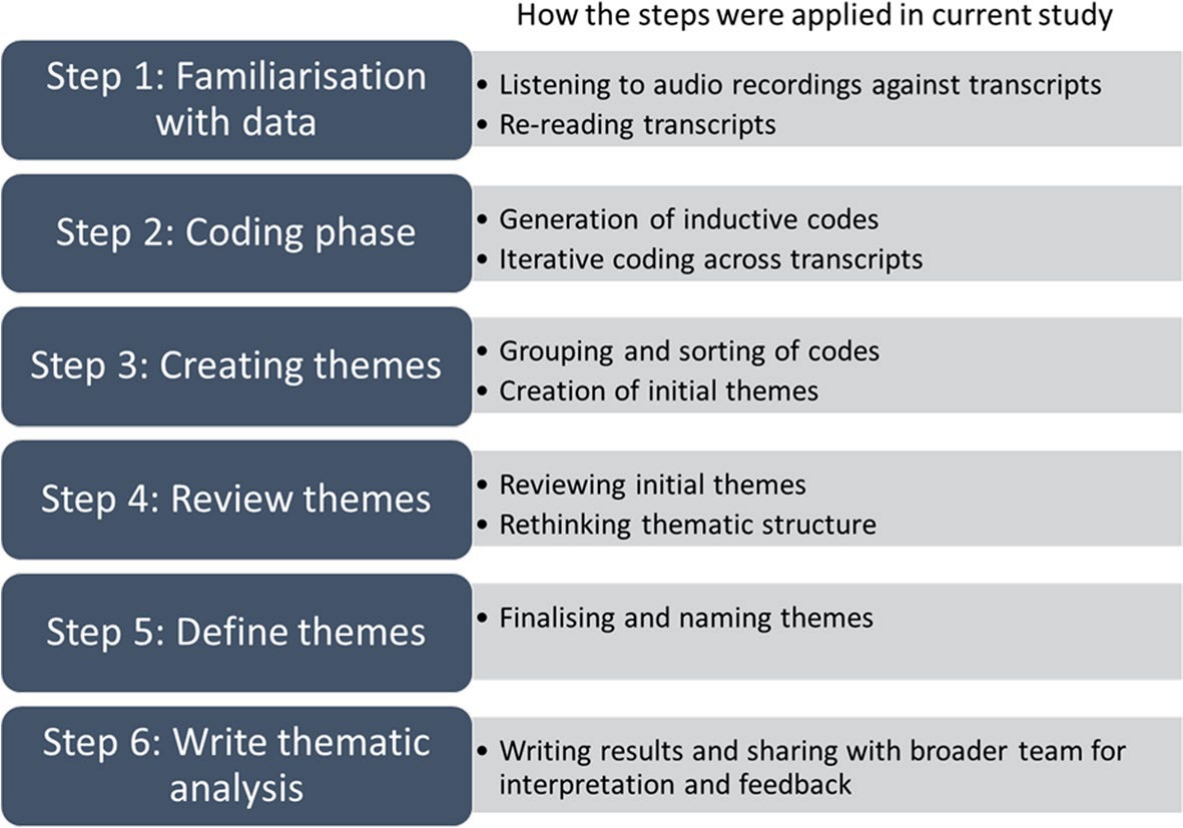
The six steps of thematic analysis [33, 34] and how they were applied in the current study

The basic principles of thematic analysis were followed, as seen in Fig. 1. This involved following the six steps of thematic analysis as laid out by Braun and Clarke [33, 34]. GM coded all transcripts, and DD coded 70% of the transcripts, to familiarise themselves with the data and the coding structure, and to incorporate alternative perspectives. NVivo 12Pro qualitative analysis software (QSR International Pty Ltd. 2018) was used for organisation and management. Team analysis meetings were held regularly (GM, DD, BJJ), and DD and GM maintained reflexive journals across all stages of data analysis to bracket assumptions, reflect on findings and document analytical queries for future discussion. This study was limited to sampling participants from the *Healthy Conversations @ Playgroup* trial, and thus data saturation did not guide recruitment. However, the themes developed through analysis were analytically robust and well supported by the data, and the team are confident that saturation of the themes presented in this article was achieved, as new data was not producing new or conflicting findings.

## Results

### Sample and participant characteristics

Twenty-eight individuals participated in this qualitative evaluation: 17 parents, 6 playgroup coordinators, and 5 peer facilitators (Additional Fig. 1). Six focus groups were conducted with parents (*n* = 2–5 per group), two focus groups were conducted with playgroup coordinators (*n* = 3 per group), and five individual interviews were conducted with peer facilitators, lasting approximately 46 min (range 36–60 min). Participants were from South Australia (*n* = 10), Western Australia (*n* = 10), and Queensland (*n* = 8). Full demographic characteristics of participants are described in Table 1. Peer facilitators were parents themselves, often familiar with the playgroup setting from personal or professional experience.

**Table 1:** Demographic characteristics of participants

Characteristic	Participants (n)
**Parents *(n* = *17)***	
State	
Western Australia	5
South Australia	6
Queensland	6
Age	
26-35 years	8
36–45 years	9
Gender	
Female/woman	17
Male/man	0
Prefer not to say	0
SEIFA quintile of household	
1 (most disadvantaged)	0
2	2
3	1
4	4
5 (least disadvantaged)	10
Main language spoken at home	
English	15/17
Non-English	2/17
Highest level of education	
TAFE/Diploma/Certificate	1/17
University undergraduate degree	6/17
University postgraduate degree	10/17
Current employment	
Casual employment	3/17
Part-time employment	5/17
Full-time employment	1/17
Full-time parent or home duties	8/17
Number of children per parent attending playgroup	
1	12
2	5
**Playgroup coordinators *(n* = *6)***	
State	
Western Australia	3
South Australia	3
Queensland	0
SEIFA quintile of playgroup	
1	3
2	0
3	0
4	0
5	3
**Peer facilitators *(n* = *5)***	
State	
Western Australia	2
South Australia	1
Queensland	2

Acronyms: SEIFA = socio-economic index for area, ranks areas in Australia to relative socio-economic advantage and disadvantage [42]

### Themes

Four main themes were derived across parent, playgroup coordinator, and peer facilitator transcript data: 1) Program strengths and challenges, 2) Setting strengths and challenges, 3) Factors impacting program delivery, and 4) Participant’s suggestions for future program delivery. All participants have been given pseudonyms. See Additional Fig. 2 for analysis coding tree.

### Theme 1: Program strengths and challenges

Within this theme, there are four subthemes: 1) Relevant, helpful conversations, 2) Reducing parenting pressures, 3) Fostering peer support, and 4) Ever-changing challenges of parenting. See Table 2 for participant quotes against each subtheme.

**Table 2:** Participant quotes for Theme 1 'Program strengths and challenges'

Subtheme	Example participant quotes
Relevant, helpful conversations	Olivia:...having that focused topic. I mean it was probably things that we would have maybe spoken about anyway amongst our peer group. You know, we’re always kind of talking about sleep and food and stuff anyway. But perhaps having that facilitator to sort of focus the conversation helped us talk about specifics. (Parent, focus group 3) Mia: And we knew that topic was coming up, so we had a chance to think about, okay, how’s our family faring with this (Parent, focus group 3) Jasmine:...it was a great way to bring up topics at playgroup... there are sometimes things we don’t naturally talk about and so by having someone like myself come in and started up conversations around topics that are, like, important to everyone actually of every age really, when it comes to healthy eating and active play. (Peer facilitator) Natalie: I guess when someone’s standing there telling you, “Oh, you know, you should do this or you should do that,” you don’t... but when you’ve got your peers telling you that, “this is the strategy that works for us,” or agreeing with what the facilitator’s saying, then you can kind of go, “okay, well maybe I will try that.” (Playgroup coordinator, focus group 2)
Reducing parenting pressures	Rachel: I felt that the facilitator... was very inclusive, was very realistic. Like realising that... you can’t have everything perfect all the time. And I thought that was, that was kind of comforting. (Parent, focus group 5) Olivia: Not necessarily like, ‘You should do this’, ‘You should do that’, because it’s never sort of one thing works with all children and families. (Parent, focus group 3) Melanie: Me personally, didn’t feel like I wanted to put any added pressure on them. (Peer facilitator) Natalie: What works for one [child] hasn’t necessarily worked for another because all kids are different (Playgroup coordinator, focus group 2)
Fostering peer support	Hazel: They [Peer Facilitator] were encouraging every parent to share their experiences..okay this is something I can try, I haven’t tried this before, so they would encourage us, ‘why don’t you try this?’ (Parent, focus group 2) Veronica: I think it’s the most important thing as a mum, having a connection with another mum so you can talk about things that are going on. (Parent, focus group 5) Kathryn: They made friendships, and got to know each other... some parents would get emotional when they were discussing these things and you could see that support coming from other members of the group, and them opening up and maybe sharing things that they hadn't shared before. That was a really positive bonding thing. (Peer facilitator) Sophia: To see all their reactions and all the relief from all of our parents that attended on those sessions... to see that everyone was normal and... invariably there was another parent that was suffering, so for that it was a relief to watch parents... they thought they were all alone, to watch that connection between the parents. (Parent, focus group 4)
Ever-changing challenges of parenting	Olivia: I think it’s hard with sleep because it’s like, especially in the first few years it’s always changing, so it’s really hard. You might think that you’ve got it all sorted and bedtime sorted, but then they’ll go through another development phase or they’ll drop a nap, and then it just gets all up in the air again. (Parent, focus group 3) Sammy: Sometimes kids change, like at the beginning of the year he may not have certain problem we talk about, but later on he started to have that and I already forgot the strategies we were talking about. (Parent, focus group 6) Grace: The eating, the sleep and the screentime, they were the really big ones that everyone sort of remembered. Whereas the movement, I don’t think it was really an issue for us because we had older children, so they were all often running, so it wasn’t really..there wasn’t any issues with that. (Playgroup coordinator, focus group 2)

### Relevant, helpful conversations

Parents described their involvement in the *Healthy Conversations @ Playgroup* program as a positive experience and described the opportunity to have conversations about relevant topics as a strength of the program. Parents commented that while it is not unusual for these topics to be discussed between parents at playgroup, they welcomed the dedicated time to have these conversations. Peer facilitators and playgroup coordinators echoed these sentiments and noted the importance of having the peer facilitator start the conversation and keep it on track. They reflected on the importance of the conversational, rather than stand-and-deliver style.

### Reducing parenting pressures 

Parents described not feeling judged by peer facilitators and other parents in the program and felt the program fostered an environment of open-mindedness. There was a shared understanding at the playgroups that what works for one family may not work for another, and that no parent is perfect. Parents also described that the program reaffirmed their choice of parenting practices and boosted their confidence. This sentiment was echoed by peer facilitators, who described intentionally approaching the conversations in a way that would not increase pressure on parents. Both peer facilitators and playgroup coordinators described the program facilitated a safe environment for parents to share their experiences and fostered an understanding that all families were different.

### Fostering peer support

Participants identified peer support as a core strength of the program, fostered by peer facilitators and other parents. The value of having a ‘peer’ facilitate the conversations, as someone who had ‘been through it’ and could speak to their own experiences, was evident across participant responses and viewed as a strength of the program. Parents provided peer support through connecting with one another and sharing their own experiences and strategies. This was particularly helpful for first-time parents who could learn from parents who had older children and had dealt with similar challenges in the past. The peer-sharing provided an opportunity for parents to identify with one another and learn from each other. The program normalised the challenges parents often face at this stage of child development, and a common shared parenting experience was fostered.

### Ever-changing challenges of parenting

Although the program topics were thought to be relevant, participants noted that some topics were of more interest than others. Some parents felt that they had already overcome the challenges associated with some topics, and others did not find the topics of relevance to their child at the time of the program. The ever-changing nature of the challenges parents face as children grow was generally cited as why topics were not always viewed as relevant for parents.

### Theme 2: Setting strengths and challenges 

Within this theme, there are three subthemes: 1) Playgroups are a suitable setting for programs supporting parents, 2) Playgroup environments can be distracting, and 3) Playgroups have varied attendance. See Table 3 for participant quotes against each subtheme.

**Table 3:** Participant quotes for Theme 2 "Setting strengths and challenges"

Subtheme	Example participant quotes
Playgroups are a suitable setting	Mia: So the kids were able to be there and around us, which was really good, because often we can’t attend a lot of things because our children aren’t welcome or it’s not easy for our children to be in that space. (Parent, focus group 3) Heather: I think with the playgroup setting, I think it's a really good idea. The reason is it's part of our routine already. We're not having to make special time (Parent, focus group 1) Kathryn: I think it certainly is a good thing that you can meet people where they're at. You're not asking them to go to a different venue to show up at a different time, in a different setting, where they're not familiar because that's hard. As a parent with young children, that's a hard thing to commit to. (Peer facilitator)
Playgroup environments can be distracting	Tahlia: Playgroup was really hard to do because it’s not in an enclosed environment. So, the parents always have to have an eye on it and so when the facilitators came to give the information and talk, we really could only give 50% of our buy-in because the other 50% is trying to track which child is doing what at the time. (Parent, focus group 5) Maria: I guess the hardest part was just because of the nature of playgroups and kids go wandering off and things, with [Peer facilitator name] trying to be able to actually get a chance to talk to everyone. (Playgroup coordinator, focus group 1) Felicity: The delivery can sometimes be difficult because of different playgroups are set up differently. (Peer facilitator)
Playgroups have varied attendance	Andrea:... every other conversation, there would be a new set of parents for me, so like my playgroup is vast, so sometimes it happens that the people who were there last week, wouldn't have been in this week... But whoever attended took it really nicely. Yeah, they were cooperative in certain ways (Playgroup coordinator, focus group 2) Deanna: And then also it was every week there were different people, so it wasn’t the same group of people which I think was hard. (Peer facilitator) Christine: because of COVID, because people were having to isolate or if they had any cold or flu symptoms, we would generally stay at home, that really prevented numbers at playgroup. A lot of participants weren't making it... and that limited the conversations we could have when there were only two or three of us there. (Parent, focus group 1)

### Playgroups are a suitable setting for programs supporting parents

Playgroups were described as a suitable setting for a child health promotion program supporting parents, as they were familiar, casual, relaxed, and safe environments. Peer facilitators and playgroup coordinators described playgroups as providing a receptive audience with the potential for broad reach in the community. Aligning with the design and intention of the program, attending playgroup was already part of parents’ routine, and therefore attendance at the program was viewed by many as convenient. Parents valued not having to make additional time to attend the program outside of their existing activities and appreciated being able to attend with their children, thus confirming the thinking behind the program design.

### Playgroup environments can be distracting 

Although playgroups were identified as a suitable and convenient setting for delivery of the program, participants acknowledged the playgroup environment as one with frequent distractions for parents and peer facilitators, due to competing demands on attention. Distractions largely came from children requesting their parent’s attention. This could pose a challenge for parents attempting to engage in the conversations, and for peer facilitators trying to facilitate the conversations. Peer facilitators and playgroup coordinators also noted the challenges of different layouts of playgroups. The way the playgroups were set-up in the space, and the way they were coordinated were noted as impacting parents’ ability to engage with the conversations.

### Playgroups have varied attendance 

Another challenge presented by the playgroup setting was the varied attendance of parents from week to week. This could impact parents’ ability to engage in conversations not just through their own attendance, but through the inconsistent presence of others. The varied attendance meant group size and dynamics changed frequently, which impacted the engagement of parents and the quality of conversations. The COVID-19 pandemic further exacerbated issues with attendance at the time.

### Theme 3: Factors that impact program delivery

Within this theme, there are five subthemes: 1) Each playgroup is unique, 2) Timing of and between sessions, 3) Group dynamics, 4) Perceived engagement with the program, and 5) Competence of peer facilitator. This theme is composed exclusively of peer facilitator and playgroup coordinator data, as it relates to how the program was run and the questions that were asked of these population groups. See Table 4 for participant quotes against each subtheme.

**Table 4:** Participant quotes for Theme 3 "Factors that impact program delivery"

Subtheme	Example participant quote
Each playgroup is unique	Kathryn:... the smaller playgroups worked better. So some playgroups [have] like 15 or more families. It was very, very hard to ever gather people together and to actually have a conversation together... So I do think the size of the playgroup made quite a difference... anywhere between five and ten was probably a nice size to actually gather together in that environment. (Peer facilitator) Andrea: So, if they [children] were all outside, we had the conversations inside, and when they were inside, we had the conversation outside. So, it was just a matter of adjusting where the kids were. (Playgroup coordinator, focus group 2) Felicity:... it worked well when you had a good playgroup facilitator [coordinator] that was really on board. Who was a little bit planned for it, I felt, that had also communicated well with their group. (Peer facilitator)
Timing of and between sessions	Kathryn: I'm a bit mixed again with the program... every second week. In some ways that was good, especially if there was families who maybe wanted to do their normal things and have a catch-up, that meant I wasn't there every week, but at the same time, I think every second week, especially if someone was away, they missed one week, then if they didn't catch me until the week after that, it would be a month since they had any contact. (Peer Facilitator) Felicity: If it was the start of term, the first week all the parents are coming together and all they want to do is chit-chat, catch up. (Peer facilitator)
Group dynamics	Maria:... the group discussion, it sort of worked on some levels. But then I think also you get people that are you know, a bit nervous to ask a question in a bigger group, like they might feel more comfortable chatting one on one. (Playgroup coordinator, focus group 1) Felicity: I think also the difference often was if the group was established as a group compared to, say a playgroup where you just felt like the parents weren't connected as much, so it was a little less open, possibly. (Peer facilitator) Melanie: The playgroup was so large so not everybody, you might have your core couple of parents that, I don’t want to say cliquey... at one particular one there was a group that they went out socialising together... I could talk to that core group but then I’d have to go and have multiple conversations with people one on one because they weren’t part of that group or didn’t feel comfortable. (Peer facilitator)
Perceived need for the program	Maria: I felt like the people who were there were already people who were, you know thinking about having a healthy diet for their kids and getting enough exercise and that sort of thing... Whereas I know that there’s other playgroups where it probably would be really, really beneficial to them to have that kind of thing. (Playgroup coordinator, focus group 1) Deanna: I had one playgroup this term where every session, the parents are so engaged and it’s like I don’t even have to be there. I just introduce the topic and they just talk about it, and they always bring up the key messages without me having to prompt them and I think, oh golly, have they read the manual or something? I don’t even need to be here. (Peer facilitator) Jasmine:You could tell there was some families who didn’t want to be part of it. That’s not why they were coming to playgroup. They were just coming to playgroup to spend time playing with their child or it was just to have I guess more of a personal conversation with a friend. (Peer facilitator) Melanie:It’s a bit hard because it’s just me seeking them and just trying to draw something out so yeah that’s probably the biggest challenges when you don’t, when you’ve got no one really interested which I would find interesting when a large number of people signed up for it but then when it came to the crunch they sort of weren’t. (Peer facilitator)
Competence of peer facilitator	Maria: But yeah I guess [Peer facilitator name] was very good in that she was flexible with how she approached it. (Playgroup coordinator, focus group 1) Melanie: I think you have to be someone who is comfortable, [it] can be quite daunting going into these new, for the first week or two you’re a new face, a new person, so it can be daunting at times but most people are really lovely and open to the discussions which is nice, makes your job easier. (Peer facilitator)

### Each playgroup is unique

It was evident from participant descriptions that each playgroup runs differently, depending on the parents, the playgroup coordinators, and the physical space and environment. Peer facilitators noted that these components impacted their delivery of the program, and how easy it was for parents to engage. From peer facilitator’s perspectives, the playgroup coordinators were integral to the program’s success. If playgroup coordinators were supportive and valued the program, and set-up the playgroup to be conducive to participation, this increased the likelihood that parents could engage.

### Timing of and between sessions

Sessions were intended to be delivered every two weeks, but because of personal illness, or COVID-19 disruptions, some peer facilitators ran sessions weekly or had longer breaks between sessions. The shorter distance between sessions was viewed positively by some peer facilitators, who found it easier to engage parents when sessions were delivered in close succession. The timing of the sessions over the year also appeared to impact parents’ engagement, with peer facilitators noting better parental participation when the program was provided further along in the school term compared to the first weeks of term, where parents were more likely wanting to ‘catch up’ after the break from playgroups over the holidays.

### Group dynamics

Peer facilitators and program coordinators described group dynamics impacting parents’ engagement in the program. Peer facilitators described conversations as easier to facilitate when parents were confident and relaxed with each other. When the dynamics were not as constructive, facilitating the conversations was more challenging, particularly when parents were not as open to sharing or contributing to discussion. Playgroups with an established group of parents led to constructive conversations, especially compared with new groups where parents were not as familiar with one another. However, peer-facilitators observed established friendship groups within a playgroup could make it difficult for those who were not part of the friendship group to contribute.

### Perceived engagement with the program

Playgroup coordinators and peer facilitators noted that a number of the parents who were involved in the program appeared to already be very confident and familiar with the topics, and thus were not as interested in participating in the conversations. Some parents were also more interested in catching up with one another or spending time with their child(ren) than engaging in the conversations. Peer facilitators noted that parents’ interest or engagement in the topics often determined how easy or challenging the conversations were to facilitate. When parents were engaged, facilitators felt they barely had to drive the conversation at all, but when parents were not interested, facilitators could feel as though they were talking to an empty room. Playgroup coordinators and peer facilitators also observed the opportunity the conversations brought to engage newer parents or those with minimal established connections at playgroup, noting that engagement could change over time from passively observing, to more actively contributing as time went on.

### Competence of peer facilitator 

Due to the dynamic nature of playgroups, it was important that the person delivering the program could adapt to each playgroup environment. The importance of peer facilitators being competent, flexible, and confident in their delivery to accommodate the playgroup environment was acknowledged in participant’s responses. As anticipated in the design of *Healthy Conversations @ Playgroup*, this was integral to program delivery, due to the varied nature of playgroups, and factors that impacted parents’ engagement in the conversations.

### Theme 4: Participant’s suggestions for future program delivery

Within this theme, there are three subthemes: 1) Who and how of program delivery, 2) Program content, and 3) Tailoring to meet playgroup needs. These are participant’s suggestions for the program based on their perceptions and experiences, and many sit in contradiction to the strengths and benefits of the program they expressed. See Table 5 for participant quotes against each subtheme.

**Table 5:** Participant quotes for Theme 4 “Participant’s suggestions for future program delivery”

Subtheme	Example participant quotes
Who and how of program delivery	Emma:... whether it would be possible to do sessions without the children, only because there was always something going on and you’d be in and out of the session, you would miss certain things because you were off doing something else because your child is screaming at you or they need to go to the bathroom. (Parent, focus group 2) Sophia:... I come home and tell my husband, you know, “We did this at playgroup, this is the conversation, this is the resources that I’m looking at,” and he sort of felt left out of the loop. (Parent, focus group 4) Deanna: The biggest thing I think would be having Playgroup [Association] on board to have it be facilitated as a sustainable program through them. So maybe it could be training some facilitators at Playgroup [Association]. (Peer facilitator)
Program content	Heather: I think even having sessions around if you've got to go and talk to certain specialists, whether it's a dietitian or a nutritionist or an OT or a speechie or whatever it might be, it's how do you ask questions of the people that are around your children all the time. (Parent, focus group 1) Tahlia: I really believe mental health for mummies is really important... I feel like everything’s been aimed at the kids which is great, but for the kids to be healthy, their mummy’s need to be healthy as well, and that they're a huge part of that. (Parent, focus group 5) Deanna: Often parents would say, “are we going to have a conversation about toilet training, or are we going to have a conversation about tooth brushing or are we going to have a conversation about behavioural issues.” (Peer facilitator) Felicity:... everyone getting a sheet while we're having the little conversation that they can physically write on just to remind themselves of some ideas and things like that, like what we've talked about. (Peer facilitator)
Tailoring to meet playgroup needs	Charlie: I think the program would be really valuable for first time parents. We you know, we still enjoyed it but I don’t think some parents with subsequent babies have the same stresses that you do the first time around. So I think maybe advertising it directly to first timers might be a good idea. (Parent, focus group 2) Natalie: The experience of having different age groups means that there is maybe someone who has been there and done it as opposed to not doing it, and then you’ve just got the facilitator basically saying, you know, going through the motions of the thing, and then you’ve got all these new mums but you don’t have any of the older mums to kind of go, “Well, this helped me or this helped.” (Playgroup coordinator, focus group 2) Felicity: Sometimes I felt like the groups were very well prepped and other times they weren't... maybe you even need a visit before you start the sessions to discuss with the group where would be the best place to do it? How would you like to do it? Would you like to do it at morning tea?... To give that group a little bit more choice, and also ownership over the discussion in a way. Not us kind of, 'Oh, well let's do it here or there'. (Peer facilitator)

### Who and how of program delivery

Due to the distracting nature of playgroups, participants suggested offering the program in a setting that more easily allows parents to concentrate, such as at a time and place away from children, or where child-supervision was provided. Participants described flexibility for delivery, including drop-in, once-off or follow-up sessions, virtual delivery of sessions, and increased opportunities for co-parent involvement. These suggestions sit in contrast to the benefits and strengths of the current delivery and setting of the *Healthy Conversations @ Playgroup* program described by participants, and it is clear that there needs to be balance between the benefits of the playgroup setting against its challenges.

These participants were asked how they envisioned long-term program delivery. Playgroup coordinators and peer facilitators suggested that support from playgroups at the jurisdiction level was required to ensure delivery of the program could be maintained through playgroups on an ongoing basis. Alternatively, they suggested other service providers who could potentially deliver the program instead. For sustainable delivery of the program, peer facilitators discussed the option for the program to be delivered by playgroup coordinators or champions.

### Program content

Participants suggested additional topics for the program. Common suggestions were behaviour management and regulation, child development, sibling relationships, speech and language development, and toileting. Parents also suggested topics related to engaging with specialists, parenting roles and support, child developmental transitions, and further information on using screen time positively. Playgroup coordinators and peer facilitators also suggested introduction of solids, parent self-care, and toothbrushing.

Peer facilitators and playgroup coordinators suggested providing more practical tips, more resources or handouts for parents to revisit, and opportunities for notetaking. However, these suggestions contradict the strength of the relaxed, informal conversation-style format of the program, which parents explicitly preferred over stand-and-deliver lecture-style programs. Participants also suggested providing practical activities for children and/or parents during the conversations that aligned with the conversation topics for each session, to keep the children busy and parents engaged.

### Tailoring to meet playgroup needs

Some participants suggested splitting the program by child age, so that only information relevant to child age and stage was being discussed. Others disputed this suggestion, as they felt this would negatively impact the peer support provided by parents with different experiences. Peer facilitators indicated that it would be helpful to have more involvement with the playgroup prior to delivering the program, to support specific tailoring of the program to individual playgroup environments and parent characteristics.

## Discussion

The study aim was to understand how the *Healthy Conversations @ Playgroup* program was experienced by parents, playgroup coordinators, and peer facilitators. Through qualitative analysis of focus group and interview data, peer support and normalising parenting challenges were found to be key program strengths. Playgroups were suitable for delivering this type of program, but the setting presented a dynamic environment that required flexibility and cooperation for successful program delivery.

Social support was a strength of the *Healthy Conversations @ Playgroup* program. The support provided by peers helped to normalise and create a shared experience of parenting. Peer support was facilitated by having parents of children of different ages and stages and having a ‘peer’ facilitate the sessions. The program helped parents feel more confident and assured in their parenting practices, which is an important aspect of parent capacity and likelihood of participating in positive parenting practices [38]. Research has shown that capacity building is an integral component of successful behaviour change [31], and without this feeling of confidence and capability, it is less likely parents would make behaviour changes at home [7, 28, 32]. Many programs in the child health promotion space provide education, advice and strategies, and the fostering of parenting support and capacity is often overlooked [7, 28, 31, 32]. For parents to be able to effectively support health behaviours in children, they need to feel supported themselves.

The *Healthy Conversations @ Playgroup* program was unique in using an existing community setting with social connection. It has been established that the health and wellbeing of caregivers, including parents, is integral to being able to care for others [39]. This sentiment was discussed by parents in the present study, “for the kids to be healthy, their mummy’s need to be healthy as well”. Parenting is challenging and too often parents feel alone in the challenges they face [40], especially first-time parents [41]. Mothers in particular bear the brunt of social expectation for their children’s health status [42, 43], and feelings of shame and stigma at not being ‘good enough’ can lead to poor outcomes for both parents and children [43]. The *Healthy Conversations @ Playgroup* program drew on the strengths of the playgroup setting, as an environment that provides social support, a sense of belonging and feelings of reassurance and validation [44, 45]. The finding that the program was able to provide this support to parents as a novel way to improve child health behaviours was reassuring, as it was an intention of the program as informed by the focus groups that preceded the program design [28].

The playgroup setting provided an optimal environment for fostering support and was considered convenient and comfortable for parents and children. Delivering the program in a setting where parents already attend is another core strength of *Healthy Conversations @ Playgroup*, and a facilitator to parental engagement in a program such as this, as it did not require transport or attendance to an additional setting [20, 46]. However, playgroups were also described as a dynamic and potentially distracting environment by all participant groups. The dynamic and distracting nature of playgroups was anticipated [28] and strategies were incorporated into the program design by having facilitators who could embrace the complexity of the environment, work constructively with enthusiastic playgroup coordinators, and engage in flexible delivery to ensure the conversations suited parents in their playgroup environment. This flexibility of delivery aligns with the emphasis on effective facilitation for successful program implementation [47]. To further strengthen program delivery in the playgroup setting, participants suggested the facilitator attend each playgroup prior to the program to understand the contexts, parent needs, and group dynamics and tailor their delivery accordingly.

While the program was viewed positively by most participants in this study, some parents felt that they were already addressing the topics presented in the *Healthy Conversations @ Playgroup* program at home. However, population-representative health survey data indicates that majority of households are still not meeting recommendations for these health behaviours [3, 4, 48–50], presenting an incongruence between what parents say they do at home, and what actually occurs. Additionally, parents valued hearing other’s experiences, but few acknowledged their role in helping others through sharing their own experiences. Playgroups pose a convenient, safe, and supportive environment for programs that aim to foster parent capacity building for promoting health behaviours in children. Program champions could be used to enhance the delivery of these programs through playgroups, helping to increase participation, reach and engagement, and motivate change.

### Strengths and considerations

This qualitative study allowed an in-depth evaluation of the *Healthy Conversations @ Playgroup* program and is one of the first qualitative evaluations of a health promotion program set in a community playgroup setting. GM, who conducted the focus groups and interviews, was independent to the program and had no part in its design or delivery, reducing the potential for social desirability bias to impact the findings. Most transcripts were coded by two members of the research team, which strengthened the interpretation of the findings and the depth of the analytical discussions with the broader team. Participants were sampled from those already attending playgroups, and had self-selected to both be a part of the *Healthy Conversations @ Playgroup* program, and this qualitative evaluation, therefore may not reflect the views and profiles of broader parent population. Finally, as this was an opportunistic evaluation of the program, many parents had received the program over 12 months prior to participating in the focus group and therefore the results presented in this study may be impacted by recall bias.

### Implications for research and practice

The *Healthy Conversations @ Playgroup* program is a first step in supporting positive child health behaviours in a playgroup setting. Many of the program strengths aligned with the intention of the program design, as informed by the focus groups with parents [28], confirming consultation with the target population as an integral step in program design. Building from these strengths, future iterations of the program could look at reach and engagement with support networks such as co-parents and other caregivers to strengthen practices at home. The use of champions to increase reach and engagement is also worth exploring. Some parents in this study did not recognise the value they had in supporting others who were struggling, which is an underutilised opportunity in the supportive environment fostered at playgroups. The scalability of the program, including how it’s delivered, and how much it’s tailored to the individual setting, is an area for future research.

## Conclusion

The *Healthy Conversations @ Playgroup* program was valued by participants and provided social support, confidence, reassurance, validation of parenting practices, and fostered normalisation and a shared experience of parenting. It provided opportunities for sharing strategies and learning from others on how to engage in autonomy supporting parenting practices at home. Playgroups are a convenient and safe setting for children and their families and are ideal for delivering health promotion programs such as *Healthy Conversations @ Playgroup*. Potential opportunities to support future delivery of the program in playgroups to help reach a broader parent population include engaging broader support networks including co-parents and grandparents, and enlisting program champions, to increase reach and engagement, motivate change, and strengthen practices at home.

### Supplementary Information

**Additional file 1.** Interview/focus group schedules. Interview/focus group schedules containing the questions that guided the focus group and interview discussions.

**Additional file 2.** Participant flow through Healthy Conversations @ Playgroup qualitative evaluation study. Figure of the flow through the study of the three population groups included in the qualitative evaluation.

**Additional file 3.** Coding tree for thematic analysis of interview and focus group data. Description: Figure of the coding tree that sits behind the results presented in the paper

## Data Availability

The datasets generated and/or analysed during the current study are not publicly available due to the nature of the data, the conditions of ethics approval, and privacy concerns, but are available from the corresponding author on reasonable request.
