# Supplementary material for: A great way to bring up health behaviour topics at playgroup: a qualitative evaluation of the Healthy Conversations @ Playgroup program
Source: BMC Public Health. 2024 Mar 25;24:890. doi: 10.1186/s12889-024-17703-x (PMC10962158; doi:10.1186/s12889-024-17703-x)
Supplement: Supplementary file 1 — Additional file 1. Interview/focus group schedules. Interview/focus group schedules containing the questions that guided the focus group and interview discussions. [file 12889_2024_17703_MOESM1_ESM.docx]

**Additional File 1 Interview/focus group schedules**

**For parents/guardians**

1. To start with, what did you think about the *program* in general?
   1. What worked well?
   2. What didn’t work so well?
2. If the program was to continue, what would you suggest we keep the same and what would you suggest we change?
   1. Why?
   2. Prompt for topics, how and where program is delivered

So just as a reminder, the Healthy Conversations program aimed to help parents with young children support each other around the topics of fussy eating, movement and active play, screen-time, and sleep.

1. Has anyone changed their parenting strategies related to any of these topics?
   1. What topic did this relate to?
   2. How? In what ways?
   3. What prompted the change?
   4. What supported the change?
   5. What components of the program supported the change? / What part did the program have in that change?
   6. Did others have different or similar experiences?
2. Were there any of these areas (fussy eating, movement and active play, screen-time, sleep) you were hoping to change, but weren’t able to?
   1. Why was this the case?
   2. In what ways could the program have supported this?
   3. Did others have different or similar experiences?

**For Playgroup coordinators and peer facilitators**

1. To start with, how did you feel about the program in general?
   1. What worked well?
   2. What didn’t work so well?
   3. What did you like/dislike about it from your role in the program?
2. How do you think the program helped parents to change their strategies?
3. What do you think needs to happen to make sure we keep the program engaging and helpful for parents? (program content)
   1. What did you find were the biggest issues with engaging parents in the current program?
   2. What solutions could you suggest to overcome these issues?
   3. Are there any other topics you think would be helpful?
4. What do you think needs to happen to keep the program ongoing in playgroups?
