# Supplementary figures and images for: A great way to bring up health behaviour topics at playgroup: a qualitative evaluation of the Healthy Conversations @ Playgroup program
Source: BMC Public Health. 2024 Mar 25;24:890. doi: 10.1186/s12889-024-17703-x (PMC10962158; doi:10.1186/s12889-024-17703-x)

## Parents

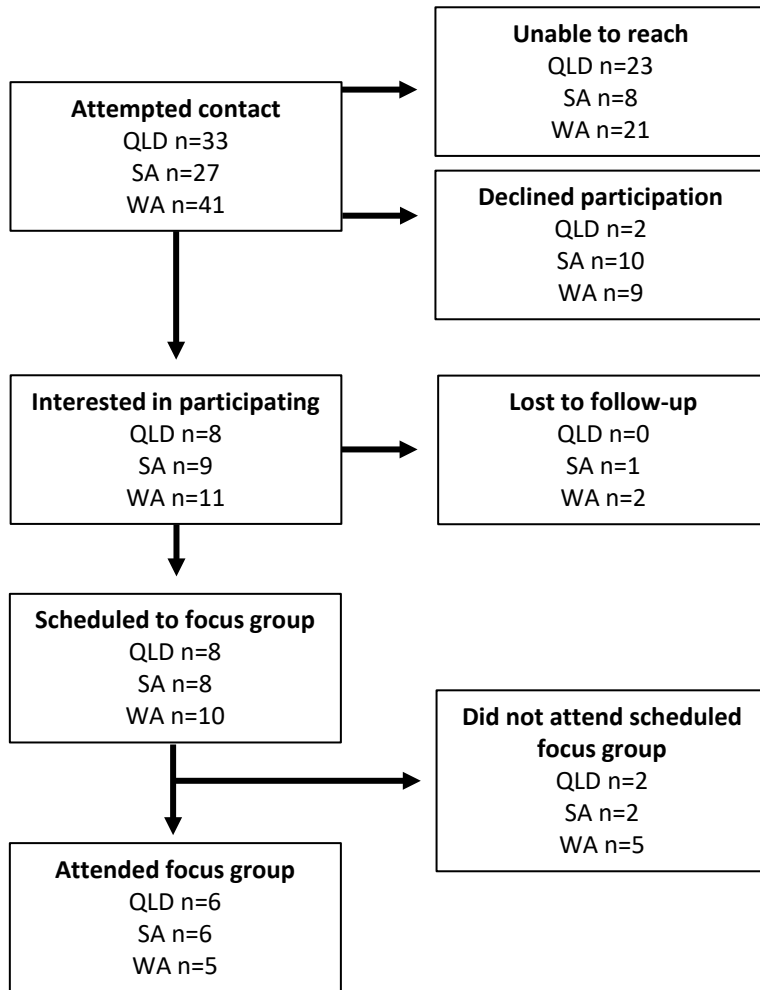

## Playgroup coordinators

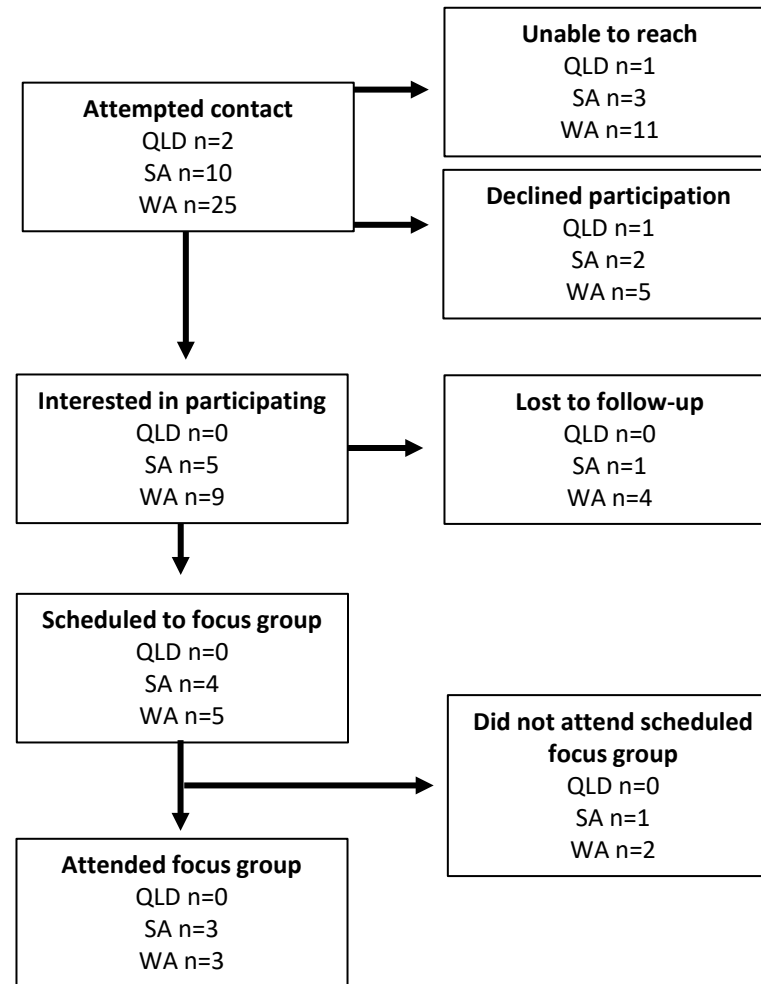

## Peer Facilitators

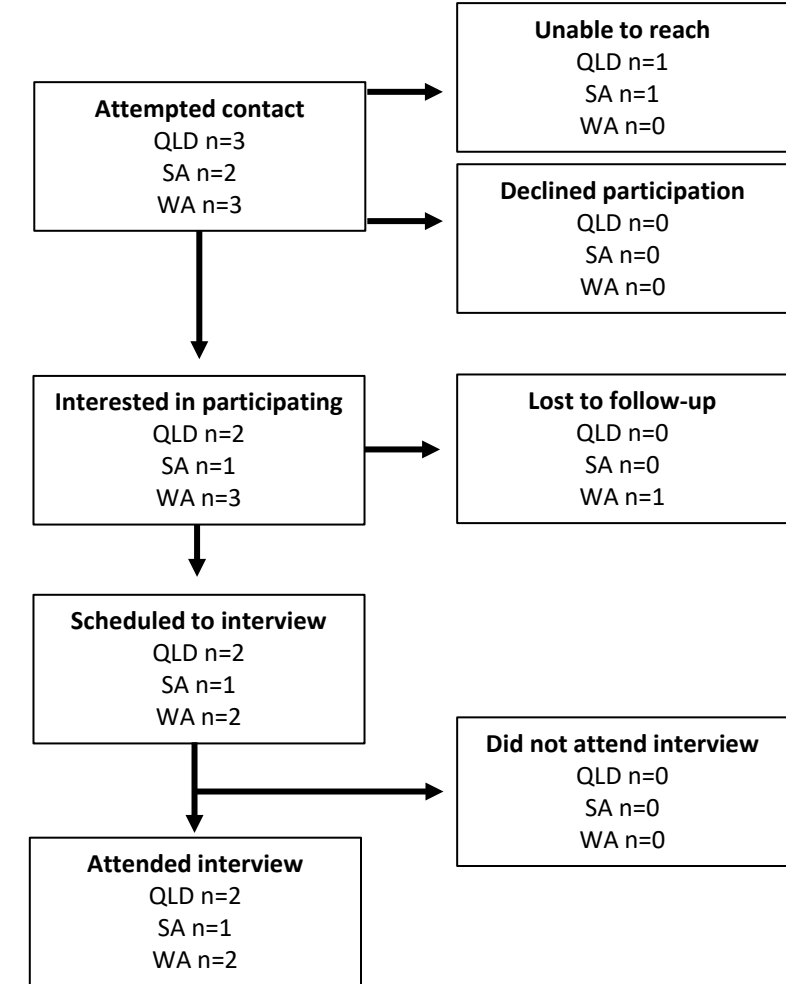

Supplement: Supplementary file 2 — Additional file 2. Participant flow through Healthy Conversations @ Playgroup qualitative evaluation study. Figure of the flow through the study of the three population groups included in the qualitative evaluation. [file 12889_2024_17703_MOESM2_ESM.pdf]

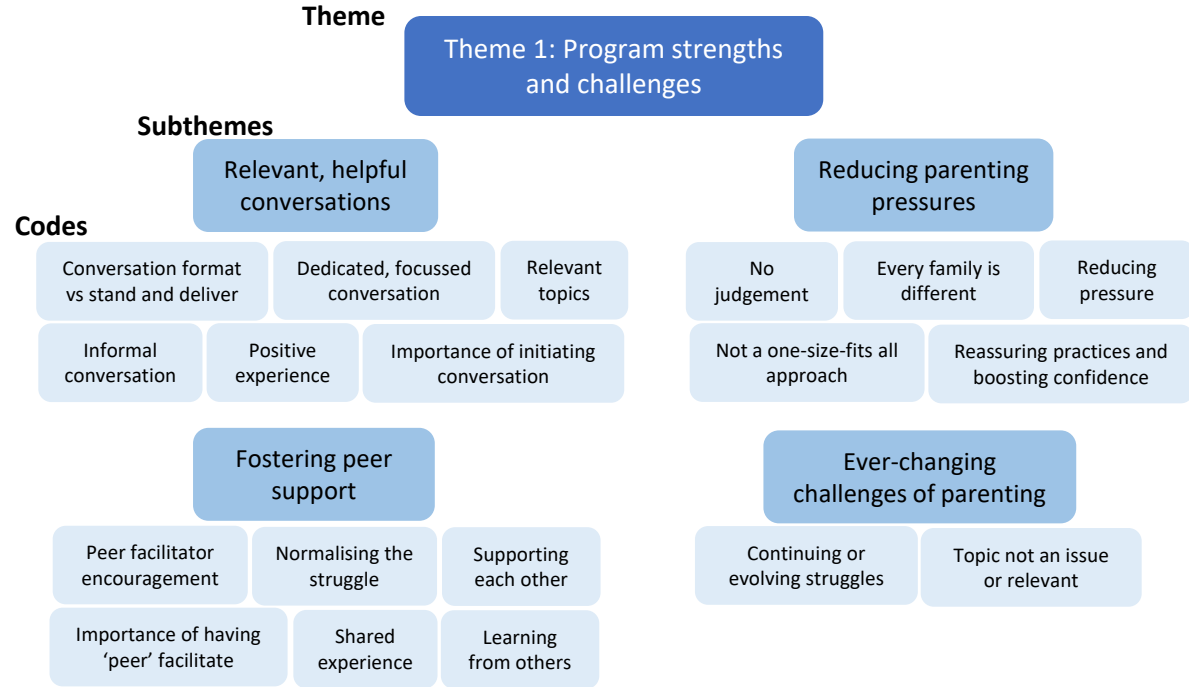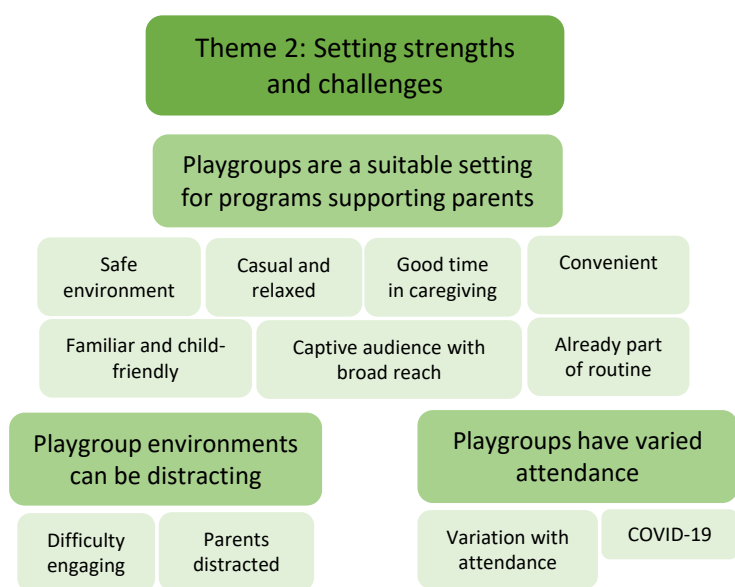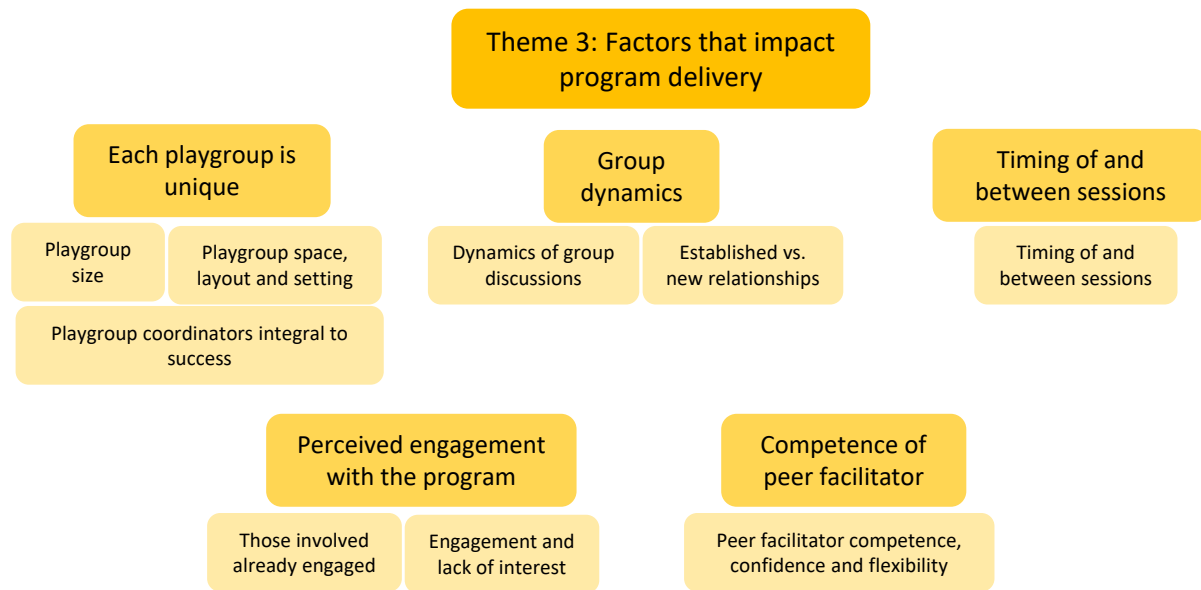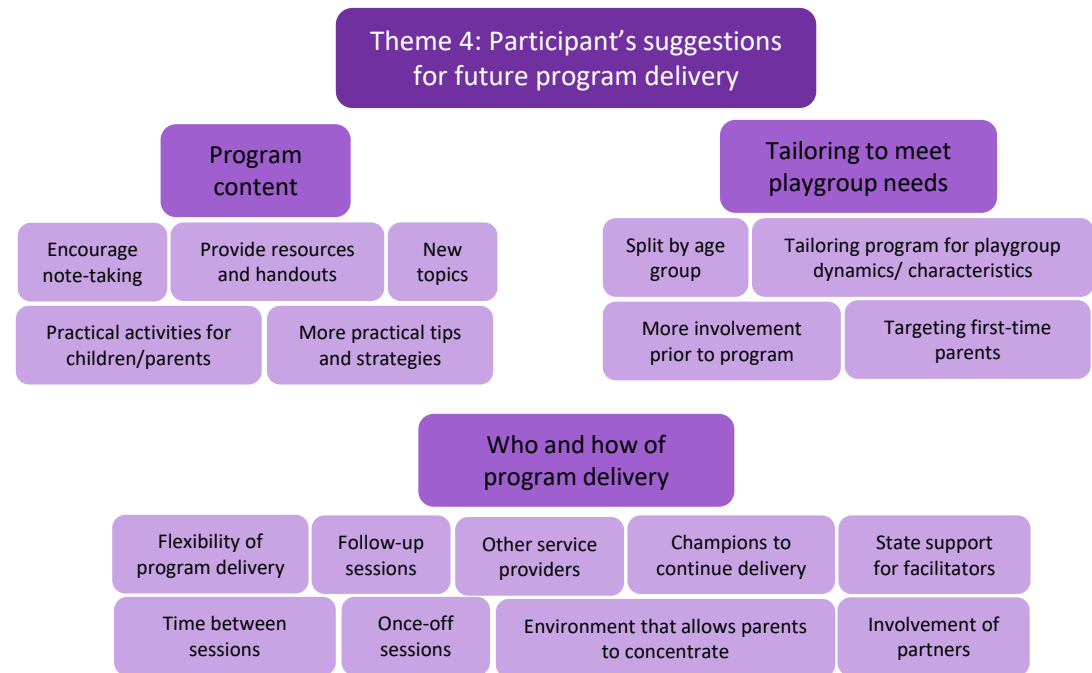

Supplement: Supplementary file 3 — Additional file 3. Coding tree for thematic analysis of interview and focus group data. Description: Figure of the coding tree that sits behind the results presented in the paper [file 12889_2024_17703_MOESM3_ESM.pdf]
